# Quercetin Dilates Retinal Arterioles via Nitric Oxide-Dependent Mechanisms in Rats

**DOI:** 10.3390/ijms27031604

**Published:** 2026-02-06

**Authors:** Asami Mori, Akihiro Sakurai, Sarina Takimoto, Kenji Sakamoto, Tsutomu Nakahara

**Affiliations:** 1 Laboratory of Medical Pharmacology, Department of Clinical & Pharmaceutical Sciences, Faculty of Pharma-Sciences, Teikyo University, 2-11-1 Kaga, Itabashi-ku, Tokyo 173-8605, Japan; p19y101228@outlook.com (S.T.); sakamoto@pharm.teikyo-u.ac.jp (K.S.); 2 Department of Molecular Pharmacology, Kitasato University School of Pharmaceutical Sciences, 5-9-1 Shirokane, Minato-ku, Tokyo 108-8641, Japan; pp11108@outlook.com (A.S.); nakaharat@pharm.kitasato-u.ac.jp (T.N.)

**Keywords:** quercetin, retinal arteriole, nitric oxide, retina, vasodilation

## Abstract

The purpose of this study was to investigate whether quercetin, a flavonoid abundantly found in onion leaves and other plant foods, induces the dilation of retinal blood vessels in rats. The time-course changes in retinal arteriolar diameter were measured using a retinal circulation evaluation system based on a high-resolution digital fundus camera developed in our laboratory. The intravenous administration of quercetin (10–100 µg/kg/min) increased the retinal arteriolar diameter in a dose-dependent manner. This vasodilatory effect of quercetin was almost completely suppressed through an intravitreal pretreatment with *N*_ω_-nitro-l-arginine methyl ester (l-NAME), a nitric oxide (NO) synthase inhibitor. In contrast, the systemic intravenous infusion of quercetin did not cause significant changes in the systemic blood pressure and heart rate. These results suggest that NO production plays an important role in the quercetin-induced dilation of retinal arterioles. Quercetin, which is abundantly present in several plant foods and possesses antioxidant properties, may be a useful agent for the prevention of various ocular diseases associated with visual impairment caused by reduced retinal blood flow.

## 1. Introduction

Glaucoma and diabetic retinopathy are major causes of visual impairment worldwide. Retinal circulatory dysfunction has been implicated in the onset and progression of these ocular diseases [1,2,3]. The neurovascular unit plays a critical role in maintaining retinal homeostasis and visual function [4,5]. Therefore, therapeutic strategies targeting the improvement of retinal circulatory dysfunction may contribute to the prevention or treatment of ocular diseases associated with visual impairment.

We have previously demonstrated in glaucoma model rats that the retinal blood flow and vasodilatory capacity decline following retinal nerve injury, and that these retinal vascular dysfunctions contribute to accelerating retinal nerve damage [6]. Furthermore, in diabetic model rats, we have reported not only retinal vascular dysfunction [7] but also abnormalities in the neurovascular unit [8]. It has also been reported that oxidative stress and inflammation are involved in retinal vascular abnormalities occurring in glaucoma and diabetes [9,10]. Based on these findings, drugs or substances with antioxidant and anti-inflammatory effects may have a protective role against retinal vascular damage in glaucoma and diabetes.

Quercetin is a flavonoid abundantly found in plant-based foods, including onions [11]. Quercetin is known for its antioxidant and anti-inflammatory effects [12], and it has been reported that quercetin dilates rat isolated mesenteric arteries, coronary arteries, and aortas [13,14,15,16,17,18]. However, the effects of quercetin on retinal vascular tone remain unclear. The purpose of this study was to clarify the effects of quercetin on the diameters of retinal blood vessels.

## 2. Results

**Figure 1:**
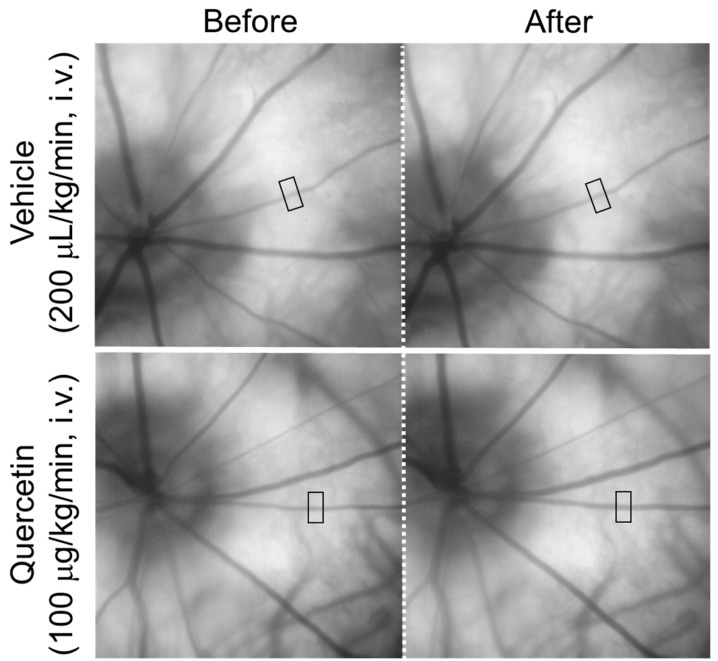
Representative fundus images before and after intravenous administration of quercetin. Representative fundus images obtained before and 10 min after intravenous infusion of quercetin (100 µg/kg/min) or its vehicle. The square indicates the region of interest used for retinal arteriolar diameter measurements (138 × 276 µm). Images show the same retinal area in each rat.

Figure 1 shows representative fundus images obtained before and after the intravenous infusion of quercetin or its vehicle. The square indicates the region used for the measurement of the retinal arteriolar diameter. In the representative images, the diameter of the retinal arteriole indicated by the square increased from 30.8 µm to 35.5 µm ten minutes after an intravenous infusion of quercetin (100 µg/kg/min), whereas an infusion of the vehicle resulted in minimal change (30.7 µm to 31.3 µm).

**Figure 2:**
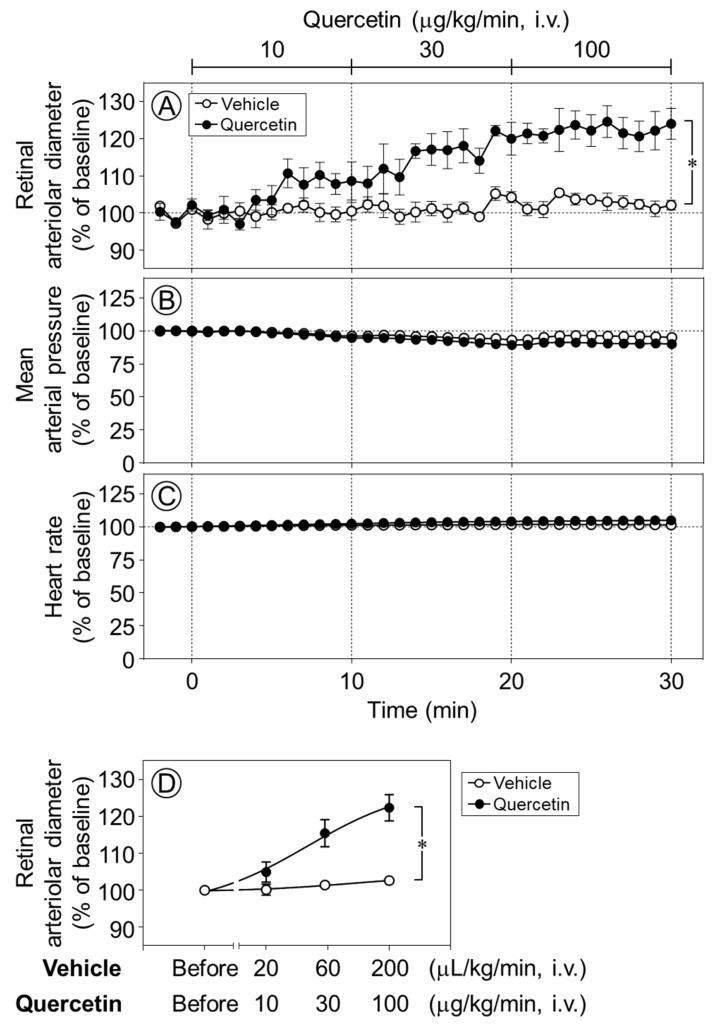
Time-course changes in retinal arteriolar diameter, mean arterial pressure, and heart rate induced by quercetin. Intravenous infusion of quercetin (10–100 µg/kg/min) significantly increased retinal arteriolar diameter compared with the vehicle (**A**). In contrast, no significant changes were observed in mean arterial pressure (**B**) and heart rate (**C**) during quercetin infusion. (**D**) Dose–response relationship of quercetin-induced retinal arteriolar dilation, in which the mean retinal arteriolar diameter during each 10-min infusion period at 10, 30, and 100 µg/kg/min was plotted against the corresponding dose derived from the time-course data shown in (**A**). Data are expressed as mean ± SEM (n = 5 per group). Open circles, vehicle; closed circles, quercetin. * *p* < 0.05.

The intravenous infusion of quercetin (10–100 µg/kg/min) increased the retinal arteriolar diameter in a dose-dependent manner, whereas the vehicle produced no notable effect. The infusion of quercetin at 30 and 100 µg/kg/min significantly dilated retinal arterioles compared with vehicle infusion (*p* < 0.05, Figure 2A,D). In contrast, neither quercetin nor its vehicle had any effect on the mean arterial pressure or heart rate (Figure 2B,C).

**Figure 3:**
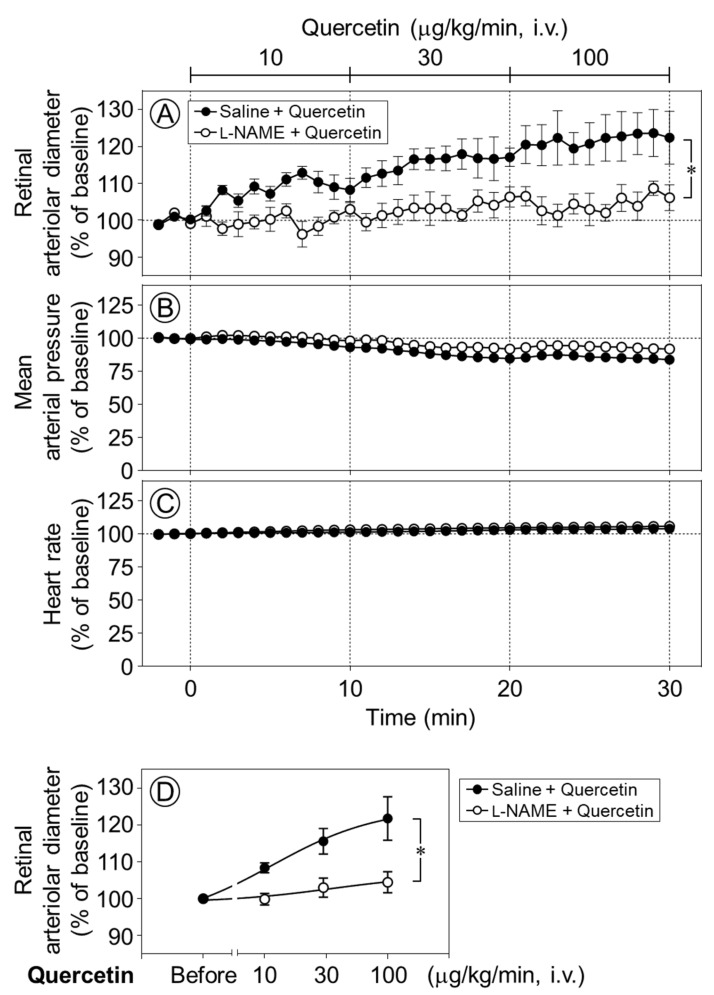
Effect of intravitreal pretreatment with l-NAME on quercetin-induced retinal arteriolar dilation. Changes in retinal arteriolar diameter in response to intravenous infusion of quercetin (10–100 µg/kg/min) were compared between saline-pretreated rats (Saline + Quercetin) and *N*_ω_-nitro-l-arginine methyl ester (l-NAME)-pretreated rats (l-NAME + Quercetin) (**A**). In contrast, mean arterial pressure and heart rate remained unchanged in both groups (**B**,**C**). (**D**) Dose–response relationship of quercetin-induced retinal arteriolar dilation, in which the mean retinal arteriolar diameter during each 10-min infusion period at 10, 30, and 100 µg/kg/min was plotted against the corresponding dose derived from the time-course data shown in (**A**). Intravitreal pretreatment with l-NAME significantly attenuated quercetin-induced retinal arteriolar dilation. Data are expressed as mean ± SEM (n = 5 per group). Closed circles, Saline + Quercetin; open circles, l-NAME + Quercetin. * *p* < 0.05.

To clarify the mechanism underlying quercetin-induced retinal vasodilation, we next examined whether nitric oxide (NO) was involved in the vasodilative effect of quercetin in the retina. Time-course changes in the retinal arteriolar diameter were evaluated following the intravenous infusion of quercetin after intravitreal pretreatment with *N*_ω_-nitro-l-arginine methyl ester (l-NAME), a NO synthase inhibitor. The quercetin-induced dilation of retinal arterioles was almost completely and significantly suppressed by l-NAME (*p* < 0.05, Figure 3A,D). Meanwhile, the intravitreal injection of l-NAME did not affect the responses of the blood pressure or heart rate to quercetin (Figure 3B,C).

In the present experiments, no significant differences were detected between the groups in the baseline values of retinal arteriolar diameter, mean arterial pressure, and heart rate prior to the administration of quercetin or its vehicle (Table 1 and Table 2).

## 3. Discussion

The intravenous administration of quercetin induced the vasodilation of retinal arterioles in rats without affecting the systemic blood pressure or heart rate. This retinal vasodilatory effect was significantly attenuated by pretreatments with a NO synthase inhibitor. These results suggest that the systemic administration of quercetin selectively dilates retinal blood vessels with little influence on systemic hemodynamics, and that NO production and release play a key role in the underlying mechanisms.

It has been reported that the topical administration of quercetin (1% solution, 50 µL) does not increase retinal blood flow in a rabbit model of ocular hypertension [19]. In contrast, the present study demonstrated that a continuous intravenous infusion of quercetin significantly increased the retinal arteriolar diameter. This discrepancy may be attributable to differences in animal species, intraocular pressure, and/or the route of quercetin administration.

In rat mesenteric arteries and the aorta, quercetin has been shown to induce endothelium-dependent vasodilation, which is mediated by enhanced NO production and/or endothelium-derived hyperpolarization (EDH) [13,15,16,20]. In rat mesenteric arteries, quercetin-induced EDH-mediated vasodilation has been reported to involve gap junctions [16]. In contrast, in rat aorta, quercetin has also been shown to induce endothelium-independent vasodilation via the activation of small conductance Ca^2+^-activated K^+^ channels [14]. Thus, quercetin can induce vasodilation through both endothelium-dependent and -independent mechanisms. In the retinal circulation, NO, prostacyclin (PGI_2_), and EDH are key factors regulating vasodilation [5]. We have previously demonstrated that acetylcholine-induced retinal vasodilation in rats involves both NO and EDH [21]. NO induces retinal vasodilation through the stimulation of PGI_2_ production [22] and activation of 4-aminopyridine-sensitive voltage-gated K^+^ (K_V_) channels [23]. In contrast, EDH mediates retinal vasodilation via gap junctions [24] and the activation of large-conductance Ca^2+^-activated K^+^ channels [7]. Although various mechanisms contribute to endothelium-dependent retinal vasodilation, the present study demonstrates that NO plays a critical role in quercetin-induced retinal vasodilation. Therefore, the downstream pathways of quercetin-induced NO production enhancement may include the stimulation of PGI_2_ synthesis and activation of K_V_ channels. Additional in vitro studies are needed to further clarify the cellular mechanisms underlying quercetin’s vasodilatory effects.

Quercetin has also been reported to suppress vasoconstriction in the rat aorta through the activation of AMP-activated protein kinase (AMPK) [25]. We have previously demonstrated that the pharmacological activation of AMPK induces retinal vasodilation via NO production and inhibits excitotoxic retinal ganglion cell death in rats [26]. In addition, quercetin has been reported to exhibit anti-inflammatory and neuroprotective effects in the brain through AMPK activation [27]. Furthermore, quercetin may exert neuroprotective effects in the diabetic retina by preventing the downregulation of neurotrophic factors and inhibiting apoptosis [28]. Based on these findings, quercetin may not only ameliorate retinal circulatory disorders but also exert protective effects on retinal neurons. Further studies are needed to clarify the potential protective effects of quercetin against retinal neurotoxicity.

In the present study, tetrodotoxin was administered to rats to suppress ocular movements that interfere with the measurement of the retinal vessel diameter, and the accompanying decrease in blood pressure was compensated by an elevating arterial pressure with methoxamine. Because these experimental conditions differ from physiological conditions, it cannot be excluded that the effects of quercetin observed in this study may not be universally applicable. However, in our previous report demonstrating that the NO donor-induced dilation of retinal arterioles was suppressed by cyclooxygenase inhibition [22], no difference in retinal vascular responsiveness was observed between rats anesthetized with thiobutabarbital and rats treated with tetrodotoxin. Therefore, we consider it likely that the vasodilatory effect of quercetin on retinal arterioles is also present under physiological conditions.

From a therapeutic perspective, topical administration is generally considered an effective route for delivering drugs to the retina. However, quercetin is widely available as a dietary supplement, and oral administration is therefore expected. In the present study, we examined the systemic effects of intravenously administered quercetin and found no significant changes in blood pressure or heart rate. The oral administration of quercetin has been reported to decrease blood pressure and improve endothelial function in hypertensive rat models, while having no hypotensive effect in normotensive control rats [29]. These findings suggest that quercetin is unlikely to induce hypotension in the absence of systemic cardiovascular disease.

Quercetin is present in several foods and is also commercially available as a dietary supplement. The regular intake of quercetin may help prevent reductions in retinal blood flow and could be useful for the prevention or treatment of ocular diseases associated with retinal circulatory impairment.

## 4. Materials and Methods

### 4.1. Animals

Male Wistar rats were obtained from The Jackson Laboratory Japan, Inc. (Yokohama, Japan). A total of 20 rats were housed under a 12 h light/dark cycle with free access to food and tap water.

All experimental procedures conformed to the Association for Research in Vision and Ophthalmology Statement Regarding the Use of Animals in Ophthalmic and Vision Research, Regulations for the Care and Use of Laboratory Animals of Kitasato University and Teikyo University. The study protocols were approved by the Ethics Committee for Animal Care and Use of Kitasato University (approval number: T04-1 and I07-1) and Teikyo University (approval number: 20-013).

### 4.2. Regents

The following reagents were used: dimethyl sulfoxide (DMSO), pentobarbital sodium, polyoxyethylene sorbitan monooleate (Tween^®︎^ 80), and tetrodotoxin (Nacalai Tesque, Kyoto, Japan); butorphanol (Vetorphale^®^; Meiji Animal Health Co., Ltd., Kumamoto, Japan); fluorescein sodium salt, methoxamine hydrochloride, *N*_ω_-nitro-l-arginine methyl ester hydrochloride (l-NAME), and quercetin dehydrate (Sigma-Aldrich, St. Louis, MO, USA); pontamine sky blue 6B, pentobarbital sodium (Tokyo Chemical Industry, Tokyo, Japan); and hydroxyethyl cellulose (Scopisol 15^®^; Senju Pharmaceutical, Osaka, Japan). Tetrodotoxin, fluorescein, methoxamine hydrochloride, l-NAME and pontamine sky blue 6B were dissolved in saline. Quercetin was dissolved in DMSO and diluted with Tween^®︎^ 80 and saline. The vehicle is a solution containing 1% DMSO, 1% Tween 80, and 98% saline.

### 4.3. Surgical Procedures

Surgical procedures were performed according to protocols previously established in our laboratory, with minor modifications [22,24]. Rats were anesthetized through the intraperitoneal administration of pentobarbital sodium (50 mg/kg)/butorphanol (2.5 mg/kg). Polyethylene catheters were placed in the jugular and femoral veins for drug administration and in the femoral artery for the continuous monitoring of systemic blood pressure and heart rate. Hemodynamic parameters were recorded using a PowerLab data acquisition system (AD Instruments, Bella Vista, Australia). Following tracheal cannulation, the animals were mechanically ventilated. To ensure stable fundus imaging at a consistent angle throughout the experimental period, eye movements were suppressed through the intravenous injection of tetrodotoxin (50 µg/kg). Because tetrodotoxin induces a reduction in systemic blood pressure, methoxamine hydrochloride (35–65 µg/kg/min, i.v.) was continuously infused to maintain adequate systemic circulation. Supplemental doses of pentobarbital sodium (10 mg/kg)/butorphanol (0.5 mg/kg) were administered as needed to maintain a stable level of anesthesia.

### 4.4. Experimental Protocols

To evaluate the effects of quercetin on the diameters of retinal arterioles, blood pressure, and heart rate, quercetin (10–100 µg/kg/min) or its vehicle was administered through continuous intravenous infusion, and the responses were observed. The dose was increased with a stepwise elevation of the drug infusion rate every 10 min. Consequently, the administered dose increased as the experimental time progressed. Quercetin was continuously administered intravenously at 10 µg/kg/min from 0 to 10 min, 30 µg/kg/min from 10 to 20 min, and 100 µg/kg/min from 20 to 30 min.

To assess the involvement of NO in the quercetin-induced retinal vasodilation, the NO synthase inhibitor l-NAME (40 nmol/eye) or its vehicle (saline, 5 µL/eye) was administered intravitreally prior to surgery. Retinal vascular responses to the subsequent intravenous infusion of quercetin were then examined. The dose of l-NAME was determined based on our previous study. Intravitreal injections were performed at least 60 min prior to the start of quercetin infusion.

### 4.5. Fundus Imaging and Measurement of the Retinal Arteriolar Diameter

Fundus imaging and quantitative analysis of retinal arteriolar diameter were conducted using methods previously established in our laboratory [22,24]. Fundus images were obtained with a digital camera (EOS7D; Canon, Tokyo, Japan) equipped with a bore scope-type objective lens (Model 01; Scalar, Tokyo, Japan). For diameter measurements, a region of interest containing a retinal arteriole (138 × 276 pixels) was cropped from the original fundus image (5184 × 3456 pixels). Retinal arteriolar diameter was then determined based on pixel-to-length conversion, with a spatial resolution of 1 µm per pixel.

### 4.6. Data Analysis

The retinal arteriolar diameter, mean arterial pressure, and heart rate were expressed as percentages (%) of the baseline level (mean values of the data obtained from time −2 to 0 min). All values are presented as the mean ± standard error (SEM). An unpaired t-test was used to compare the baseline values between the two groups. Responses to quercetin between the groups were compared using linear mixed models followed by the Tukey–Kramer HSD test (JMP Pro version 15, SAS Institute Inc., Cary, NC, USA). Differences were considered statistically significant when the *p*-value was less than 0.05.

## Figures and Tables

**Table 1:** Baseline values of retinal arteriolar diameter, mean arterial pressure and heart rate in rats.

Group	Retinal Arteriolar Diameter (μm)	Mean Arterial Pressure (mmHg)	Heart Rate (Beats/Min)
Vehicle (n = 5)	29.7 ± 0.7	104 ± 2	386 ± 24
Quercetin (n = 5)	30.7 ± 1.4	108 ± 3	395 ± 7

Values are means ± SEM. These values were measured just before starting the infusion of vehicle or quercetin.

**Table 2:** Baseline values of retinal arteriolar diameter, mean arterial pressure and heart rate in rats.

Group	Retinal Arteriolar Diameter (μm)	Mean Arterial Pressure (mmHg)	Heart Rate (Beats/Min)
Saline + Quercetin (n = 5)	41.9 ± 0.7	114 ± 1	375 ± 14
l-NAME + Quercetin (n = 5)	44.4 ± 1.8	111 ± 1	362 ± 9

Values are means ± SEM. These values were measured just before starting the infusion of quercetin.

## Data Availability

The data presented in this study are available on figshare at https://doi.org/10.6084/m9.figshare.30962489.

## Abbreviations

The following abbreviations are used in this manuscript:

l-NAME	*N*_ω_-nitro-l-arginine methyl ester
NO	Nitric oxide
EDH	Endothelium-derived hyperpolarization
PGI_2_	Prostacyclin
K_V_	Voltage-gated K^+^
AMPK	AMP-activated protein kinase
DMSO	Dimethyl sulfoxide
i.v.	Intravenous
